# The impact of Kahramanmaraş (2023) earthquake on adolescents: Exploring psychological impact, suicide possibility and future expectations

**DOI:** 10.1017/gmh.2024.90

**Published:** 2024-11-27

**Authors:** Veysel Kaplan, Mehmet Emin Düken, Rabia Kaya, Muhammad Alkasaby

**Affiliations:** 1 Faculty of Health Sciences, Nursing Department, Harran University, Şanlıurfa, Turkey; 2 Faculty of Health Sciences, Nursing Department, Harran University, Şanlıurfa, Turkey; 3 Faculty of Health Sciences, Nursing Department, Harran University, Şanlıurfa, Turkey; 4 Centre for Global Mental Health, London School of Hygiene & Tropical Medicine, London, United Kingdom

**Keywords:** adolescents, mental health, earthquake, psychological symptoms, suicide, future expectation, Turkey

## Abstract

Earthquakes and other disasters caused by natural hazards have a significant impact on the mental health and well-being of children and adolescents. This study aimed to investigate the psychological symptoms, suicide probability, and future expectations among adolescents affected by the Kahramanmaraş-centered earthquake in Türkiye. A total of 704 individuals participated in the study. We conducted a cross-sectional study using the Brief Symptom Inventory, Suicide Probability Scale and Future Expectation Scale. The mean age of participants was 15.27 ± 1.39. Participants lost up to 10 of their relatives and up to 4 of their nuclear families due to the earthquake. The study showed a strong positive correlation between psychological symptoms and suicide probability and a strong negative correlation between psychological symptoms and future expectations among adolescents. Additionally, losing family members or relatives was associated with increased psychological problems. Earthquake-related issues such as lack of food, shelter and security, and education disruption should be addressed to mitigate the mental health impact of the disaster. Additionally, mental health and psychosocial support services should be made available for adolescents and their families in the earthquake-affected regions.

## Impact statement

In February 2023, the world witnessed one of the most devastating earthquakes in the twenty-first century that hit Türkiye and Syria and led to the death of over 55,000 people. This disaster was followed by other earthquakes in Morocco and Afghanistan in the same year, which drew attention to the mental health impact of outbreaks, especially on vulnerable groups such as children and adolescents. Our study examines the impact of earthquakes on the mental health of a sample of adolescents living in the earthquake-affected regions in Türkiye. Our study found that adolescents are experiencing several psychological problems, such as anxiety, depression, and somatization. This was associated with a negative impact on their future expectations and a higher risk of suicide. In addition, Some factors such as adolescent’s young Age, loss of relatives, and prolonged stay under the rubble were associated with increased psychological problems experienced by adolescents, negatively affected their future expectations, and increased their risk of suicide.

These findings urge caregivers, health and education personnel, and to give special attention to the psychological needs of adolescents. Those who are involved with adolsents in different settings (e.g. home, school) should be trained in addressing adolescents’ psychological needs. Humanitarian organizations and others responding to earthquakes and other emergencies should develop and implement targeted interventions that address the psychosocial issues identified in this study.

## Introduction

An earthquake is “a violent and abrupt shaking of the ground, caused by movement between tectonic plates along a fault line in the earth’s crust. Earthquakes can result in ground shaking, soil liquefaction, landslides, fissures, avalanches, fires and tsunamis” (World Health Organization, 2023). These events, which can cause widespread structural and socio-economic destruction, are life-threatening, unexpected, unpredictable, and uncontrollable (Maslovaric et al., 2017). The two major earthquakes of magnitude 7.8 and 7.6 that hit Türkiye and Syria are considered among the deadliest earthquakes in the twenty-first century (Naddaf, 2023; Kurt et al., 2023). One of the most significant impacts of earthquakes is the health impact. Studies have shown that exposure to disasters such as earthquakes, floods or hurricanes causes a wide range of mental problems, especially among vulnerable groups such as women and children (Norris et al., 2002; Başoğlu et al., 2004; Zhang et al., 2010; Özdemir et al., 2015; Ahmed et al., 2023; Kaplan et al., 2024). Adolescents are more susceptible to the psychological impacts of traumatic events (Kaplan et al., 2021; Maeda et al., 2009; Hızlı et al., 2009; Zhang et al., 2010). A study showed that mental health conditions persist in children and adolescents even 4 years after a major earthquake (Hızlı et al., 2009). Various psychological problems such as acute stress reactions, adjustment disorder, depression, anxiety disorders, and post-traumatic stress disorder (PTSD) may occur in adolescents after a disaster (Tanaka et al., 2016). They may also experience other behavioral problems such as academic failure, crime and substance abuse. This may be due to their dependency on adults in terms of care, shelter, transportation, and protection and their lack of the experience, skills, and resources to independently meet their mental and behavioral health needs (National Commission on Children and Disasters, 2010).

One of the serious mental problems that adolescents face after traumatic events such as disasters is suicidal ideation (Yang et al., 2005; Stratta et al., 2014). Suicide is the fourth leading cause of death in individuals aged 15–29 (World Health Organization, 2021). Psychological consequences of natural disasters, such as PTSD (Sahin et al., 2007), anxiety and depression (Tang et al., 2018), have been studied, but little is known about suicide. Existing studies mostly focus on adults (Stratta et al., 2012; Xu et al., 2018; Guo et al., 2018), with relatively few studies focusing on adolescents (Tang et al., 2018; Gerstner et al., 2020). Therefore, there is a need to examine the possibility of suicide after natural disasters, especially because adolescents have poor coping skills during challenging life events, can be more affected by traumatic events, and disruptions in their daily routines lead to leads to many challenges (Krug et al., 1998).

Adolescence is a critical period in which social and psychological growth occurs and expectations for the future are determined (Hazen et al., 2008). Traumatic life events such as earthquakes and epidemics experienced during adolescence can cause serious problems such as anxiety, fear, loneliness, and decreased interest in the environment and the future (Bozkurt et al., 2020; Jefsen et al., 2021; Kaplan et al., 2021; Liu et al. 2020). A study reported that individuals who exhibit suicidal behaviors also show a lack of positive expectations for the future (MacLeod et al. 1997). In their study, Yılmaz et al. (2005) stated that earthquake experience plays an important role in expectations for the future. People who have previously experienced earthquakes have reduced expectations for the future, which is associated with the destructive and negative effects of earthquakes on their lives.

Several studies examined the psychological impact of crises on adolescents, and especially post-traumatic stress (Başoğlu et al. 2004; Ben-Ezra et al., 2015; Ben-Zur and Almog, 2013; Ge et al., 2020; Gerstner et al., 2020). However, to our knowledge, no study examined the relationship between psychological symptoms, suicide probability, and future expectations among adolescents affected by earthquakes. The earthquakes in Syria and Türkiye in February 2023 were followed by several earthquakes in Morrocco, Afghanistan, and Nepal. This emphasizes the importance of studying the mental health impact of earthquakes, especially on vulnerable groups such as children and adolescents. Our study aims to assess psychological symptoms, suicide probability, and future expectations among adolescents affected by the recent earthquake in Türkiye and associated factors in order to inform future interventions targeting populations affected by the earthquake.

## Methods

### Study design and setting

The present study is a descriptive, cross-sectional, and correlational study. It was conducted to investigate the psychological symptoms, suicide probability, and future expectations of adolescents who were affected by the Kahramanmaraş-centered earthquake. The study was conducted 3 months after the earthquake in Adıyaman, Kahramanmaraş, and Hatay where the earthquake had the most impact.

### Participants and sampling

The target population was adolescents who were affected by the earthquake and were present in temporary camps and settlements, assembly areas, and aid distribution centers. Due to logistical issues and the lack of a register for all individuals in these places, we used a convenience sampling approach. Individuals who met the inclusion criteria were included in the study.

#### Inclusion criteria

Adolescents who met the following criteria were included in this study;

- willing to participate,
- living in the three cities most affected by the earthquake: Adıyaman, Kahramanmaraş, and Hatay,
- Age is between 12–18,
- have no problem in terms of reading or understanding the data collection tools, and
- have not been diagnosed with any mental disorders in the past.

### Data collection

The data was collected through face-to-face interviews between 01.06.2023 and 01.07.2023. A form providing information about the study and its purpose and consent details was shared with the adolescents and their caregivers. In addition, informed consent was obtained from the participants’ parents. Each interview took approximately 30 min. The data collection form consists of the following components:

***Personal Information Form:*** The form was created by the researchers in light of the literature (Ge et al., 2020; Gerstner et al., 2020) to determine the various sociodemographic (Age, education level, etc.) and some contextual factors (number of family member and relatives/friends lost due to the earthquake, time under the debris etc.) that may impact adolescents psychologically.

***Brief Symptom Inventory (BSI):*** The inventory was developed by Derogatis (1992) to identify psychological symptoms experienced by individuals. Şahin and Durak (1994) have conducted a validity study of its Turkish version. This 5- point Likert-type inventory has 53 items with five subscales: anxiety, depression, negative self-concept, somatization, and hostility. The inventory items are scored as follows: 0 (not at all), 1 (slightly), 2 (moderately), 3 (very), and 4 (extremely). The highest possible score is 212, and the lowest is 0. This inventory is suitable for adolescents and adults. Higher scores on the BSI indicate more severe psychological symptoms. The Cronbach’s alpha coefficient (internal consistency) for the Turkish version of BSI was 0.96. (0.88 for depression, 0.87 for anxiety, 0.87 for negative self, 0.75 for somatization, and 0.76 for hostility).

***Suicide Probability Scale (SPS):*** The SPS scale is a 36-item, self-report measure that assesses suicide risk in adults and adolescents. It was developed by Cull and Gill (1988). Individuals are asked to rate the frequency of their subjective experience and past behaviors using a four-point Likert-scale ranging from “none” to “all of the time”. The scale has a score range of 36–144. Higher SPS scores indicate higher suicide probability. Eskin (1993) adapted this scale to Turkish, and assessed its reliability and validity. The Cronbach alpha value of this scale is 0.87, and test–retest reliability coefficient was 0.98.

***Future Expectations Scale for Adolescents (FESA):*** The scale was developed by McWhirter and McWhirter (2008) and adapted into Turkish by Tuncer (2011). It consists of four dimensions (work and education, marriage and family, religion and society, and health and life) with a total of 25 items. All items start with “When I Become an Adult”, and each item is scored between one (I absolutely do not believe) and seven (I absolutely believe). The FESA score ranges between 25–175. Higher FESA scores mean higher future expectations. The Cronbach Alpha coefficient scale is 0.925 for the original scale and 0.928 for the Turkish version.

### Data analysis

We conducted reliability and multicollinearity analyses. First, percentage, mean, median, standard deviation, min and max values were calculated to summarize the participants’ characteristics and calculate the scale score averages. Additionally, the relationship between SPS, FESA, and BSI was examined using correlation. Finally, the study examined the effect of BSI on SPS and FESA using the linear regression model and enter-step method. Results were considered significant if the p-value ≤0.05.

### Ethical permission

The ethics committee permission was obtained from the Clinical and Human Research Ethical Committee of Harran University (Protocol Number: E-76244175-050.01.04-226642) and the research conforms to the provisions of the Declaration of Helsinki Helsinki in 1995 (as revised in Brazil 2013).

The study information and consent form was shared with the adolescents and their parents before conducting the survey. The form contained information about the present study and participant’s rights. It explained that participants could withdraw from the study at any time without any negative consequences and that confidentiality will be considered throughout the study.

## Results

A total of 738 adolescents who fullfil elgiblity criteria were approached, and 704 agreed to participate in the study. The mean Age of the participants was 15.27 ± 1.39. Participants lost a median of one person from their nuclear family and four people from their relatives due to the earthquake. The mean time they spent under rubble was 8.04 ± 19.4 h, which is less than the mean time parents spent under rubble (24.46 ± 34.7 h for fathers and 20.76 ± 31.8 h for mothers). Table 1 summarizes participants’ characteristics and contextual factors that may affect adolescents’ mental health.

**Table 1.** Distribution of individual characteristics of adolescents

	Mean ± SD	Median (min–max)
Age	15.27 ± 1.39	15 (13–18)
Number of family members who died	1.14 ± 1.01	1 (0–4)
Number of relatives who died	4.09 ± 2.42	4 (0–10)
Time under the debris (in hours)	8.04 ± 19.4	0 (0–120)
Waiting time for the father to emerge from the debris	24.46 ± 34.7	0 (0–120)
Waiting time for the mother to emerge from the debris	20.76 ± 31.8	0 (0–160)

Table 2 summarizes SPS, FESA, and BSI scales and BSI subscales. The BSI mean score of the adolescents was 139.1 ± 45.39, the mean score of anxiety was 33.55 ± 11.43, the mean score of depression was 31.1 ± 10.42, the mean score of negative self was 31.7 ± 10.5, the mean score of somatization 23.7 ± 7.82 and mean score of hostility was determined as 19.04 ± 6.2. It was determined that the SPS mean score of the adolescents was 93.47 ± 8.4 and the FESA mean score was 87.17 ± 18.66.

**Table 2.** Total mean scores of women from scales and sub-scales

	Mean ± SD	Median (min–max)
BSI	139.1 ± 45.39	159 (47–191)
Anxiety	33.55 ± 11.43	39 (7–47)
Depression	31.1 ± 10.42	35 (7–45)
Negative Self	31.7 ± 10.5	36 (10–46)
Somatization	23.7 ± 7.82	27 (4–34)
Hostility	19.04 ± 6.2	22 (5–27)
SPS	93.47 ± 8.4	96 (74–110)
FESA	87.17 ± 18.66	80 (60–131)

Table 3 presents the results of correlation analyses of the mean scores of the different scales included in this study. There was a strong negative correlation between the FESA and both SPS (r = −0.778) and BSI (r = −0.891) mean scores, meaning that adolescents’ future expectations decrease with the increase of SPS and BSI scores. Additionally, There was a strong positive correlation between SPS and BSI mean scores (r = 0.783), which means that suicide probability in adolescents increases with the increase of psychological symptoms such as depression, anxiety and negative self.

**Table 3.** Correlation between adolescent’s SPS, FESA, BSI, and BSI subscales mean scores

		FESA	BSI	Anxiety	Depression	Negative self	Somatization	Hostility
SPS	r	−0.778**	0.783**	0.784**	0.779**	0.764**	0.753**	0.738**
	p	0.000	0.000	0.000	0.000	0.000	0.000	0.000
FESA	r		−0.891**	−0.886**	−0.880**	−0.870**	−0.864**	−0.846**
	p		0.000	0.000	0.000	0.000	0.000	0.000

** Correlation is significant at the 0.01 level (2-tailed).

Table 4 shows the correlation analysis between participants’ sociodemographics and the mean scores of the scales included in this study. There was a weak negative correlation between age and SPS and BSI mean scores and a weak positive correlation with the FESA mean score. By contrast, other variables showed a positive correlation with SPS and BSI mean scores and a negative correlation with the FESA mean score. All correlations between sociodemographics and scales mean scores range from weak (±0.1) to moderate (±0.69).

**Table 4.** Correlation analysis between various adolescents’ sociodemographics and mean scores of scales/subscales

		SPS	FESA	Anxiety	Depression	Negative Self	Somatization	Hostility	BSI
Age	r	−0.185**	0.170**	−0.156**	−0.133**	−0.155**	−0.137**	−0.105**	−0.144**
	p	0.000	0.000	0.000	0.000	0.000	0.000	0.006	0.000
Number of family members who died	r	0.625**	−0.667**	0.589**	0.579**	0.583**	0.570**	0.563**	0.591**
	p	0.000	0.000	0.000	0.000	0.000	0.000	0.000	0.000
Number of relatives who died	r	0.636**	−0.644**	0.685**	0.689**	0.665**	0.678**	0.672**	0.693**
	p	0.000	0.000	0.000	0.000	0.000	0.000	0.000	
Time under the debris	r	0.240**	−0.159**	0.153**	0.168**	0.174**	0.171**	0.162**	0.169**
	p	0.000	0.000	0.000	0.000	0.000	0.000	0.000	0.000
Waiting time for the father to emerge from the debris	r	0.260**	−0.358**	0.447**	0.477**	0.451**	0.473**	0.445**	0.469**
	p	0.000	0.000	0.000	0.000	0.000	0.000	0.000	0.000
Waiting time for the mother to emerge from the debris	r	0.473**	−0.398**	0.365**	0.345**	0.357**	0.339**	0.360**	0.361**
	p	0.000	0.000	0.000	0.000	0.000	0.000	0.000	0.000

** Correlation is significant at the 0.01 level (2-tailed).

The regression model created between the adolescents’ SPS and the BSI subscales was statistically significant (F:101.953, *p <* 0.001) (Table 5). A one-unit increase in the anxiety and depression scores of adolescents causes an increase of 0.262 and 0.226 units in the SPS mean scores (*p <* 0.001), respectively. This model explains 61.9% of the SPS scores of adolescents.

**Table 5.** Regression model established for SPS of adolescents

	B(%95)	Beta	t	p	Zero-order	Partial
(Constant)	95.824 (89.611–102.038)		30.280	***p <* 0.001**		
Anxiety	0.262 (0.12–0.405)	0.357	3.611	***p <* 0.001**	0.784	0.137
Depression	0.226 (0.069–0.382)	0.280	2.835	**0.005**	0.779	0.108
Negative Self	0.037 (−0.096–0.17)	0.047	0.548	0.584	0.764	0.021
Somatization	0.079 (−0.283–0.086)	0.082	1.048	0.295	0.753	−0.040
Hostility	0.013 (−0.369–0.043)	0.02	1.550	0.122	0.738	−0.059

B(%95): Non-standardized Coefficients, Beta: Standardized Coefficients, Adj R^2^:0.619, F:101.953, *p <* 0.001, S.E: 4.972.

Similarly, the regression model created between adolescents’ FESA mean score and BSI subscales mean scores was statistically significant (F:119.896, *p <* 0.001) (Table 6).

**Table 6.** Regression model established for FESA of adolescents

	B(%95)	Beta	t	p	Zero-order	Partial
(Constant)	168.689 (159.722–177.656)		36.937	***p <* 0.001**		
Anxiety	−0.558 (−0.789 - –0.326)	−0.342	−4.731	***p <* 0.001**	−0.886	−0.178
Depression	−0.413 (−0.668 - –0.158)	−0.231	−3.179	***p <* 0.001**	−0.880	−0.121
Negative Self	−0.248 (−0.465 - –0.031)	−0.140	−2.246	**0.025**	−0.870	−0.086
Somatization	−0.211 (−0.512–0.09)	−0.088	−1.375	0.170	−0.864	−0.053
Hostility	−0.14 (−0.196–0.477)	−0.047	0.818	0.413	−0.846	0.031

B(%95): Non-standardized Coefficients, Beta: Standardized Coefficients, Adj R^2^:0.801, F:119.896, *p <* 0.001, S.E: 8.116.

A one-unit decrease in adolescents’ anxiety and depression scores leads to a 0.558 and 0.413 unit increase in their the FESA mean scores (*p <* 0.001), respectively. Similarly, a one-unit decrease in adolescents’ negative-self scores provides a 0.248-unit increase in their the FESA mean scores (*p =* 0.025). This model explains 80.1% of the FESA scores of adolescents.

## Discussion

This study highlights the negative impact of earthquakes on adolescents’ mental health and future expectations. Adolescents affected by the earthquake that hit Türkiye in February 2023 experienced several psychopathological and psychological symptoms, such as depression, anxiety, somatization, low self-perception, and aggression. These symptoms increased suicide probability among adolescents and negatively impacted their future expectations.

The average BSI score was 139.1 ± 45.39, which is significantly high compared to other studies on similar populations. In a study conducted among adolescent workers in Istanbul (15–18 years old), the average BSI score was 57.6 ± 36.0, which is less than half of the average BSI score calculated in our study (Örnek and Esin, 2018). Several studies showed that mental health-related symptoms, such as PTSD symptoms, depression, anxiety, somatization, insomnia, psychotic experiences, are frequent among adolescents impacted by natural disasters such as earthquakes, floods and tsunamis (Aksu and İmrek, 2023; Cénat et al., 2020; Goenjian et al., 2011; Marthoenis et al., 2019; Sharma et al., 2021). Several factors contribute to disasters’ negative impact on adolescents’ mental health, including the destruction of the individual’s home, loss of family members or relatives, and many adverse conditions such as the lack of a safe shelter, clean water and food, and experiencing aftershocks (Aksu and İmrek, 2023; Ben-Ezra et al., 2015; Hong and Efferth, 2016).

Our study showed a positive correlation between higher scores of BSI and the loss of family members and relatives. Adolescents who participated in this study lost a median of one person from their nuclear family and four people from their relatives due to the earthquake. Existing literature shows that experiencing loss may cause serious mental problems characterized by negative emotions such as loneliness, fear, sadness, anxiety, helplessness, meaninglessness or feeling worthless (Çubuk, 2020; Kübler-Ross and Kessler, 2014; Özel and Özkan, 2020; Wilson, 2014). Additionally, they were trapped under the rubble for an average of 8 h and waited for a long time for the rescue of their parents. Such a situation, which represents a threat to the lives of adolescents and their families, would have a significant impact on their mental health.

Suicide probability scores of adolescents in this study were relatively high (93.47 ± 8.4) compared to other studies conducted with similar populations. In a study of adolescents aged between 14 and 18 years old in Türkiye the Average SPS score was 69.05 ± 16.06 for males and 72.44 ± 16.62 for females (Cenkseven-Önder, 2018). Several other studies indicate that natural disasters such as earthquakes pose serious risks in terms of suicidal ideation, suicide attempts and suicide intention in adolescents (Stratta et al., 2014; Tang et al., 2018; Ying et al., 2015). Such traumatic events cause significant disruption in adolescents’ environment and social support networks, and hence reduce their ability to cope with the situation. Our study found a positive correlation between losing family members and relatives and suicide probability. Perceived social support was found to be a protective factor against suicide (Cenkseven-Önder, 2018). Therefore, losing family members and relatives and the disruption of social networks due to earthquakes may increase the probability of suicide, as shown in this study.

Regarding future expectations, our study showed lower scores of FESA among adolescents impacted by the earthquake. Additionally, there was a strong negative correlation between FESA and all BSI subscales, such as depression, anxiety and negative self. In a study conducted in Türkiye among high school students, the average FESA score was (130.81 ± 31.94) which is significantly higher than the average FESA score in our study (87.17 ± 18.66). Literature shows that traumatic events such as earthquakes, natural disasters, pandemics, and war negatively impact adolescents’ views and expectations for the future (Ben-Zur and Almog, 2013; Commodari and La Rosa, 2020; Saupe et al., 2019). An earthquake is a traumatic event in which an individual loses his/her relatives or safe areas such as home and as a result, his/her perception of life may change (Artar, 2003). Furthermore, emotional or behavioral changes experienced by adolescents due to the earthquake play a key role in the future expectations of the individual (Artar, 2003). In contrast, positive future expectations can have a positive impact on mental health. A study found that adolescents’ future expectations influence their health and psychological well-being in adulthood (Kim and Kim, 2020). Another study among children affected by HIV/AIDS found that positive future expectations mitigate the negative impact of traumatic events on mental health (Zhang et al., 2009).

### Limitations

The study has a few limitations. Initially, the data collection took place 3–4 months after the earthquake, which may carry the risk of pathologizing adolescents’ normal responses to the crisis. Furthermore, the study sample was taken from makeshift settlements established for individuals who lost their homes, which may contribute to elevated psychological symptoms within the sample, and may not be representative of the entire population affected by the crisis. It is worth noting that individuals diagnosed with a mental health condition were excluded, hence, this study did not capture the exacerbation of symptoms among those with pre-existing mental health conditions due to the earthquake. With the utilization of a non-probability sampling method, the study’s outcomes are only applicable to the participants in the study. Nevertheless, this study’s findings could still serve as an indicator of the substantial impact of the crisis on the mental health of adolescents and guide efforts to mitigate this impact.

#### Recommendations

Based on this study results and our experience of this disaster and similar emergencies, we recommend the following:

- Address earthquake-related issues that may impact adolescents’ mental health such as lack of food, shelter and security, and education disruption,
- Scale up mental health services for adolescents and their families in the earthquake-affected regions,
- Provide social protection for adolescents who lost their families and caregivers,
- Train teachers, social workers, health providers, and others working with adolescents on understanding and supporting adolescents’ mental health needs,
- Develop school-based mental health interventions to address mental health issues among adolescents including suicide and negative future expectations,
- Conduct longitudinal studies following adolescents over time after the earthquake to understand long-term mental health trajectories,
- Assess the effectiveness of different psychosocial interventions in addressing earthquake-related mental health issues and suicide risk among adolescents.

## Conclusion

Earthquakes have a significant impact on the psychological well-being of adolescents. In addition to increasing psychological symptoms, our study shows an increase in suicide probability and negative future expectations among adolescents affected by the earthquake. Given the role of the family and social networks in supporting adolescents’ well-being, losing family members and relatives was associated with poor psychological outcomes among adolescents. Social protection should be provided for those who lost their caregivers. Earthquake-related issues such as lack of food, shelter and security, and education disruption should be addressed to mitigate the mental health impact of the disaster. Additionally, mental health and psycho-social support services should be made available for adolescents and their families in earthquake-affected regions.

## Data Availability

The data analyzed during this study are available from the corresponding author on reasonable request.
