# Supplementary material for: The impact of Kahramanmaraş (2023) earthquake on adolescents: Exploring psychological impact, suicide possibility and future expectations
Source: Glob Ment Health (Camb). 2024 Nov 27;11:e115. doi: 10.1017/gmh.2024.90 (PMC11704369; doi:10.1017/gmh.2024.90)
Supplement: Peer review history

# Editor report: Recommendation: The impact of Kahramanmaraş (2023) earthquake on adolescents: Exploring psychological impact, suicide possibility and future expectations — R0/PR2

**DOI:** 10.1017/gmh.2024.90.pr2

**Affiliations:** Department of Global Health and Social Medicine, Harvard Medical School, United States

No accompanying comment.

# Editor report: Decision: The impact of Kahramanmaraş (2023) earthquake on adolescents: Exploring psychological impact, suicide possibility and future expectations — R0/PR3

**DOI:** 10.1017/gmh.2024.90.pr3

**Affiliations:** London School of Hygiene & Tropical Medicine, United Kingdom of Great Britain and Northern Ireland

No accompanying comment.

# Editor report: Recommendation: The impact of Kahramanmaraş (2023) earthquake on adolescents: Exploring psychological impact, suicide possibility and future expectations — R1/PR5

**DOI:** 10.1017/gmh.2024.90.pr5

**Affiliations:** Department of Global Health and Social Medicine, Harvard Medical School, United States

No accompanying comment.

# Editor report: Decision: The impact of Kahramanmaraş (2023) earthquake on adolescents: Exploring psychological impact, suicide possibility and future expectations — R1/PR6

**DOI:** 10.1017/gmh.2024.90.pr6

**Affiliations:** London School of Hygiene & Tropical Medicine, United Kingdom of Great Britain and Northern Ireland

No accompanying comment.
